# A qualitative analysis of control-value appraisals, positive achievement emotions, and EFL performance in a Chinese senior high school context

**DOI:** 10.3389/fpsyg.2022.986684

**Published:** 2022-09-15

**Authors:** Weihua Yu, Hanwei Wu, Wanzhu Zhao

**Affiliations:** ^1^School of English Language, Zhejiang Yuexiu University, Shaoxing, China; ^2^School of English, Jilin International Studies University, Changchun, China

**Keywords:** control-value appraisals, positive achievement emotions, EFL performance, qualitative study, Chinese high school students

## Abstract

Based on the control-value theory (CVT), this study qualitatively investigated the relationship between control-value appraisals, achievement emotions (mainly enjoyment, pride, and hope), and English-as-a-foreign-language (EFL) performance, and explored other antecedents of achievement emotions in addition to control-value appraisals. Data were collected from six Chinese high school students through two semi-structured interviews and one focus group discussion. With thematic analysis, data were analyzed under the framework of the CVT using NVivo 11.0. Results indicate that high perceived control, high perceived extrinsic, and intrinsic values were interactively associated with enjoyment, pride, and hope. Low perceived control, high perceived extrinsic value, and low perceived intrinsic value were interactively associated with fewer positive achievement emotions (only hope) and more negative achievement emotions like fear. High perceived control, high perceived extrinsic value, and low perceived intrinsic value were interactively associated with fewer positive achievement emotions (only pride), and more negative achievement emotions like anguish. Besides, positive achievement emotions were positively associated with EFL performance, while negative achievement emotions were negatively associated with EFL performance. Moreover, factors inside the classroom, factors outside the classroom, and personal factors were found to be the antecedents of achievement emotions in EFL learning.

## Introduction

Emotions are pervasive in foreign language learning and closely associated with foreign language learning achievement. The studies on the emotions experienced by foreign language learners emerged in the 1970s. However, in the past five decades, researchers have mainly probed the effect of negative emotions, especially anxiety, on foreign language learning (Chastain, 1975; Horwitz et al., 1986; Horwitz, 2010; Liu and Huang, 2011). Until recently, with the advances of positive psychology and its introduction to the field of second language acquisition, researchers have begun to move beyond negative emotions and toward positive emotions, so as to investigate foreign language learning emotions from a holistic perspective (Macintyre and Gregersen, 2012; Dewaele and Alfawzan, 2018; Elahi Shirvan et al., 2020; Shao et al., 2020a; Kruk, 2022).

In addition, emotioncy (emotion + fluency), a newly-developed concept, also elucidates the substantial role of emotions in foreign language learning. Emotioncy is defined as the emotions induced by multisensory combinations, which can relativize cognition and then affect learning achievement (Pishghadam et al., 2013). Naturally, a combination of different numbers of senses results in different levels of emotioncy (Pishghadam et al., 2013). Emotioncy comprises seven levels, i.e., null, audio, visual, kinesthetic, inner, arch, and mastery. And it is divided into four categories, i.e., avolvement, exvolvement, involvement, and metavolvement (Pishghadam et al., 2019). Each emotioncy level builds upon the previous level, and higher emotioncy levels benefit learning better. By implication, some degree of emotions is reasonable to be implemented in foreign language teaching to help learners to increase their emotioncy levels.

Although the significance of holistic emotions in foreign language learning has been recently recognized and expounded by scholars from diverse perspectives, a framework that can be used to integrate and analyze emotions in foreign language learning is needed. The control-value theory (CVT) provides such a framework. Put forward by Pekrun (2006), the CVT addresses origins, situational specificity, functions, regulation, and relative universality of achievement emotions. The CVT (Pekrun and Perry, 2014) posits that control-value appraisals act as proximal determinants to affect academic achievement, with achievement emotions as mediators. In detail, control appraisal refers to individuals’ perceived controllability over achievement activities or achievement outcomes. Value appraisal comes to individuals’ perceived importance of achievement activities or achievement outcomes. Intrinsic value and extrinsic value are distinguished by the CVT. The former is related to achievement activities or achievement outcomes themselves rather than their instrumental functions. For instance, a person has an affection for English because he finds learning English enjoyable for its own sake. The latter is associated with the instrumental functions of achievement activities or achievement emotions for obtaining desired consequences. For example, a person attaches importance to learning English because he regards English as a promoter to his career.

Achievement emotions (Pekrun and Perry, 2014) are defined as the affective arousal directly related to achievement activities. Achievement emotions mainly comprise positive emotions like enjoyment, hope, and pride, as well as negative emotions like anger, anxiety, hopelessness, and boredom. According to the CVT, different levels of control-value appraisals can solely or interactively instigate different achievement emotions, thereby exerting effects on academic achievement. For instance, low perceived value can elicit boredom, while high perceived control and high intrinsic value can induce enjoyment, and low perceived control and high perceived value can trigger anxiety. Overall, control-value appraisals have a positive correlation with positive emotions and a negative correlation with negative emotions. Besides, the CVT (Pekrun and Perry, 2014) also reveals that the effect of achievement emotions on academic achievement is achieved by a series of motivational and cognitive mechanisms. More specifically, positive emotions can make contributions to better academic performance through reinforcing motivation, interest, and self-regulation; increasing available cognitive resources; and promoting the adoption of more flexible and in-depth learning strategies in academic settings, whereas negative emotions result in worse academic performance through weakening motivation, interest, and self-regulation; decreasing available cognitive resources and promoting the adoption of more rigid and shallow learning strategies in academic settings.

Notwithstanding the critical importance of control-value appraisals as proximal antecedents, other distal antecedents that have an impact on achievement emotions and then have a ripple impact on academic achievement are also recognized by the CVT (Pekrun and Perry, 2014), mainly including autonomy support (Tsai et al., 2008; Simonton et al., 2021), goal structures (Murayama and Elliot, 2009), cognitive quality (Cordova and Lepper, 1996), and so forth. In addition, the CVT also demonstrates the subject-specificity (Pekrun and Perry, 2014) of achievement emotions. In other words, the variety and intensity of achievement emotions may differ in diverse subjects, thus exerting a different influence on academic achievement in different subjects. By implication, it is reasonable to conduct subject-specific studies. Therefore, the CVT provides an effective framework for researchers to have explorations into emotion, motivation, cognition, and academic achievement in a specific subject from the overall perspective.

Based on the CVT, a multitude of studies have been conducted in recent years. Nevertheless, the majority of these studies have centered on mathematics (Boehme et al., 2017; Putwain et al., 2018, 2021; Held and Hascher, 2022) and science (Mercan, 2020; Beymer et al., 2021; Henschel, 2021). Apart from the numerous studies pertaining to second language emotions (Shao et al., 2020a; Yu, 2021), it was not until recent years that completely CVT-based studies started to emerge. To the best knowledge of the authors, there have been several prototypical relevant studies. For instance, within the framework of the CVT, Shao et al. (2020b), recruiting 550 Chinese university students as participants, quantitatively found that perceived control and perceived value were positively correlated with positive emotions and foreign language performance, and negatively correlated with negative emotions, and negative focal emotions played a mediating role between control-value appraisals and foreign language performance. Besides, Yang et al. (2021) qualitatively explored Chinese English learners’ achievement emotions and their antecedents in online courses during the period of COVID 19 and found that students experienced various emotions such as enjoyment, relaxation, anxiety, guilt, boredom, and hopelessness and that four antecedents existed in online English courses, namely Internet connection, workload outside the classroom, students’ self-regulation of learning behavior, and learning environment. Moreover, Li (2021), with the employment of mixed methods, collected quantitative data from 2002 university students, along with qualitative data from 12 university students and 12 university teachers, concluding that different control-value appraisals solely and interactively predicted boredom and more complex associations existed between control-value appraisals and boredom.

According to the above completely CVT-based studies, we can sum up several features. First, most relevant studies have concentrated on mathematics and science instead of the field of foreign language learning. Besides, most CVT-based studies on foreign language learning have focused on control-value appraisals and achievement emotions but failed to have incorporated foreign language performance. In addition, a quantitative approach is usually employed for confirmatory research, while a qualitative approach is generally adopted for exploratory research. Finally, in most (not all) CVT-based studies on foreign language learning, university students rather than high school students are usually selected as participants, though high school students, especially Chinese high school students, probably experience stronger emotions as they are all faced with Gaokao (unified Chinese national university entrance examinations).

Therefore, in the light of the features shared by most existing literature and the extensive research on negative second language emotions (particularly anxiety; Dewaele and Alfawzan, 2018; Russell, 2020; Aslan and Thompson, 2021), we conducted a qualitative study to investigate the relationship between control-value appraisals, positive achievement emotions (pride, enjoyment, and hope) and EFL performance with Chinese senior high school students as participants under the framework of the CVT, as well as to explore some distal antecedents of achievement emotions in addition to control-value appraisals with the hope to offer empirical support for emotional interventions in the Chinese senior high school students’ EFL learning. Specifically, the following three research questions guided our study.

RQ1: In the Chinese high school EFL context, how are control-value appraisals associated with achievement emotions?
RQ2: In the Chinese high school EFL context, how are achievement emotions associated with EFL performance?
RQ3: In the Chinese high school EFL context, what are the other antecedents of achievement emotions in addition to control-value appraisals?

## Materials and methods

### Participants

For the convenience of sampling (Dörnyei, 2007), we recruited six senior high school students from Zhejiang, China as the participants of our study. Specifically, the inclusion criteria for the participants are as follows: (1) the participants were voluntary for the study; (2) the participants were from the same class and taught by the same English teacher; (3) two participants were selected in each range of English grade (approximately 80, 100, 120 in the midterm English exam); and (4) both male and female participants were selected since experienced achievement emotions may differ from genders. The midterm English exam was a test with a broad variety of items including multiple choices, a cloze-test, fill-in-the-blanks, and essay writing (range 0–150; the Cronbach’s α = 0.759) so that the exam could effectively reflect the participants’ EFL performance. Before the research, the six participants were required to read carefully the informed consent that detailed the purpose, procedure, anonymity, and confidentiality. The present study was approved by the Ethics Committee of Jilin International Studies University.

### Instrument and procedures

Our study consisted of two interviews and one focus group discussion. The participants’ grade in the final English exam was set as the indicator of their EFL performance. More specifically, we conducted our first interview 1 month earlier than the final English exam to examine the participants’ perceived control and perceived value of their EFL learning. The first interview includes three questions for control and two questions for value (e.g., the question “How much does your effort made in EFL learning get returned?” is for control). We carried out our second interview 1 week before the final English exam to assess their achievement emotions since they would experience stronger emotions for the approaching English exam. The second interview comprises 3 questions for each emotion (enjoyment, hope, and pride. For example, the question “Is it often enjoyable for you to attend English class and why?” is for enjoyment). We implemented our focus group discussion 1 week after the final English exam to explore the other antecedents of achievement emotions in EFL learning in addition to control-value appraisals. For example, we asked the question: “Are there any factors affecting your perceived importance to EFL learning or your emotions in EFL learning?” in the focus group discussion. At the same time, the participants’ grade in the final English exam was also collected. The two interviews and the focus group discussion were conducted in Chinese to avoid language barriers and were recorded with the permission of the participants. It is noteworthy that other emotions in addition to enjoyment, pride, and hope might also be elicited in the interviews. We transcribed all the records into text through repeatedly and carefully listening and checking.

### Data analysis

The data collected from the two interviews were analyzed under the CVT framework, and the data from the focus group discussion were analyzed with thematic analysis using NVivo 11.0. Thematic analysis (Braun and Clarke, 2006) refers to six phases, i.e., familiarization, initial coding, searching for themes, reviewing themes, definition of themes, and reporting. All data shared the same coding procedures, as they are all text sources. In detail, the control-related data from the first interview (control-value appraisals) were analyzed according to the number of positive and negative answers. For example, participants with positive answers like “My effort put into EFL learning always gets returned” rather than negative answers like “I think my English grade is determined by my luck rather than my proficiency” would be evaluated to have high perceived control. Similarly, participants with negative answers rather than positive answers would be evaluated to have low perceived control. The value-related data were analyzed according to their statements. For instance, participants with the statements indicating or not indicating instrumental utility like “I think English is important because it’s required by Gaokao” or “I do not think English is important because we do not use English for communication in China” would be evaluated to have high or low perceived extrinsic value. By the same token, participants with the statements showing high intrinsic value like “I love English so much because I find it interesting,” or low intrinsic value like “I dislike English because I find it boring” would be evaluated to have high or low perceived intrinsic value. The data from the second interview (achievement emotions) were also analyzed based on the number of positive and negative answers. Take enjoyment for example, participants with positive answers like “I often feel enjoyable in English class” rather than negative answers like “I feel vexed in English class” would be evaluated to have high enjoyment. Similarly, participants with negative answers rather than positive answers would be evaluated to have low enjoyment. The data of the participants’ hope and pride were analyzed in the same way as enjoyment. With reference to the data from the focus group discussion (exploration into other antecedents), the remarks showing antecedents of achievement emotions in EFL learning such as “my English teacher was so humorous, which makes me enjoyable in English class” would be coded. Moreover, the factors influencing the participants’ value of EFL learning would also be coded, since the CVT assumes that distal antecedents can affect achievement emotions by means of influencing control-value appraisals (Pekrun and Perry, 2014).

Data validation was checked by considering credibility, consistency, and reliability (Guba and Lincoln, 1994). To increase credibility, we reviewed the initial themes carefully, conducted member checking, and validated the emerging codes and categories through discussions until an agreement was reached. To create reliability, we invited one expert to have the second reviewing process and revised part of the codes to the point on which we and the expert both agreed. To ensure generalizability, we recruited both male and female participants and recorded our research steps. To achieve consistency, we reviewed the interviews, the focus group discussion, and the codes again and again.

## Results

### Control-value appraisals and achievement emotions of the participants

Two themes emerged from the data of the first interview, namely perceived control and perceived value. Perceived value includes two subthemes of intrinsic and extrinsic values. Four themes emerged from the data of the second interview, including enjoyment, pride, hope, and negative emotions.

#### Control-value appraisals

The evaluation of the six participants’ control-value appraisals is presented in Table 1. More specifically, regarding perceived control over EFL learning, participants 2, 3, 4, and 6 were considered to have high perceived control since they had more positive answers about control. For example, participant 4 mentioned:

**Table 1:** Participants’ control-value appraisals.

Participant	Control		Value		Evaluation
	Positive	Negative	Intrinsic	Extrinsic	
1	0	3	Low	High	LC + LI + HE
2	3	0	High	High	HC + HI + HE
3	3	0	High	High	HC + HI + HE
4	3	0	High	High	HC + HI + HE
5	0	3	Low	High	LC + LI + HE
6	3	0	Low	High	HC + LI + HE

Positive, numbers of positive answers; Negative, numbers of negative answers; L, low; H, high; C, control; I, intrinsic value; E, extrinsic value.

*“The effort that I made in EFL learning is paid off. After all, my English grade is better than most of my classmates”* (Participant 4—Interview 1).

Besides, participants 2, 3, 4, and 6 also replied that those uncontrollable and unchangeable factors could not determine their English grade and luck almost does not have any effect on their English grade at all. On the contrary, participants 1 and 5 were regarded to have low perceived control since they had more negative answers. The two participants all clearly expressed their lack of control over EFL learning. For example, participant 1 mentioned:

“*I find it quite easy to forget the vocabulary I have memorized so many times, which is totally beyond my control. Maybe some people have language talent. But I don’t think I have that talent”* (Participant 1—Interview 1).

As to the perceived value of EFL learning, all participants were evaluated to have high extrinsic value with their statements emphasizing instrumental utility. For example, they all mentioned that the critical importance of English is highlighted by Gaokao. Participants 2, 3, and 4 were evaluated to have high intrinsic value since they had diverse positive statements to express their strong affection for English. For instance, participant 4 stated:

*“I love English so much. I find English so interesting when watching English speeches and amazingly dubbed English clips”* (Participant 4—Interview1).

However, participants 1, 5, and 6 were considered to have low intrinsic value with their negative remarks. Participants 1 and 5 expressed their dislike for English. For example, participant 1 mentioned:

*“I don’t like English very much. It’s boring, especially when it comes to English grammar”* (Participant 1—Interview1).

Participant 6 showed her indifferent attitude toward English since she said:

*“I don’t care much about whether I like English or dislike it. For me, what I think about is just to enhance my English grade for Gaokao”* (Participant 6—Interview 1).

#### Achievement emotions

The evaluation of the six participants’ achievement emotions is presented in Table 2. More specifically, participants 2, 3, and 4 were regarded to have more positive emotions (high enjoyment, high pride, and high hope) with their more positive answers about the three emotions. Take enjoyment for example, they all found English class and learning English-related knowledge enjoyable, and regarded English tests as an enjoyable challenge. For instance, participant 2 mentioned:

**Table 2:** Participants’ achievement emotions.

Participant		1	2	3	4	5	6
Enjoyment	Positive	0	3	3	3	0	0
	Negative	3	0	0	0	3	3
Hope	Positive	2	3	3	3	2	1
	Negative	1	0	0	0	1	2
Pride	Positive	0	3	3	3	0	3
	Negative	3	0	0	0	3	0
Negative emotions		Fear	/	/	/	Fear	Anguish
Evaluation		LE + HH + LP	HE+HH + HP	HE+HH + HP	HE+HH + HP	LE + HH + LP	LE + LH + HP

Positive, numbers of positive answers; Negative, numbers of negative answers; L, low; H (first), high; E, enjoyment; H (second), hope; P, pride.

*“I am very happy in English class. It is my only solace in my monotonous life. …I feel very happy for learning new knowledge. I think an English exam is an enjoyable challenge when it is difficult because learning English is an enjoyable thing”* (Participant 2—Interview 2).

On the other hand, participants 1, 5, and 6 were considered to have fewer positive emotions. Specifically, participants 1 and 5 experienced low enjoyment and low pride with their more negative answers about enjoyment and pride. For example, they were both reported to have gained almost no enjoyment in learning English-related knowledge and English class, and show no pride in their English proficiency, as indicated in the following excerpt:

*“I don’t feel joyful when learning English-related knowledge…My English level is lower than most of my classmates…My ability in dealing with English tests is not good. I often find reading comprehension difficult”* (Participant 1—Interview 2).

Moreover, although participants 1 and 5 experienced the fear of answering questions asked by the English teacher in the English class, both of them were evaluated to have high hope with their more positive answers about hope. For example, they both held a positive belief that their English would be improved through their hard work. Participant 6 was considered to have experienced low enjoyment and low hope with her more negative answers about enjoyment and hope. For example, she stated that she did not feel enjoyable when learning English-related knowledge, and clearly delineated the strong negative emotions she suffered from in English tests, as shown in the following excerpt:

*“I don’t feel happy when learning something new. I feel very anguished in English exams. I don’t even want to take English exams”* (Participant 6—Interview 2).

Besides, she also expressed her low hope of English learning as follows:

*“I don't think my English will be improved in the future, because I don’t think I will use English after Gaokao. I want to major in medicine, so I don't think English will be needed”* (Participant 6—Interview 2).

Furthermore, participant 6 was evaluated to have high pride with her more positive answers about it, since she stated her English level, her performance in English and her ability in handling English tests were better than most of her classmates.

### Control-value appraisals, achievement emotions and EFL performance

The CVT (Pekrun, 2006; Pekrun and Perry, 2014) posits that achievement emotions play a mediating role between control-value appraisals and academic achievement. In other words, control-value appraisals can affect achievement emotions, and achievement emotions can influence academic performance. Therefore, our analysis was divided into two parts, namely the association between control-value appraisals and achievement emotions and the association between achievement emotions and EFL performance.

#### Association between control-value appraisals and achievement emotions

We made Table 3 including the participants’ control-value appraisals, positive emotions (enjoyment, pride, and hope), and negative emotions. According to the assumption of the CVT that different control-value appraisals can interactively elicit different academic emotions, we analyzed the relationship between these variables by comparing their similarities and differences.

**Table 3:** Participants’ control-value appraisals and achievement emotions.

Participant	Control	Value		Enjoyment	Pride	Hope	Negative emotions
1	Low	High E	Low I	Low	Low	High	Fear
2	High	High E	High I	High	High	High	/
3	High	High E	High I	High	High	High	/
4	High	High E	High I	High	High	High	/
5	Low	High E	Low I	Low	Low	High	Fear
6	High	High E	Low I	Low	High	Low	Anguish

E, extrinsic; I, intrinsic.

The CVT assumes that different combinations of different levels of control-value appraisals can induce different achievement emotions. Based on the assumption, by analyzing participants 2, 3, and 4, we found that perceived high control, high extrinsic, and intrinsic values of EFL learning were interactively associated with positive achievement emotions in EFL learning. According to participants 1 and 5, we found that low perceived control, high perceived extrinsic value, and low perceived intrinsic value were interactively associated with fewer positive achievement emotions (only hope), and more negative achievement emotions like fear. Based on participant 6, we found that high perceived control, high perceived extrinsic value, and low perceived intrinsic value were interactively associated with fewer positive achievement emotions (only pride), and more negative achievement emotions like anguish.

More specifically, enjoyment was closely associated with intrinsic value. By comparing participants 6 and participants 2, 3, and 4 (different in perceived intrinsic value), we could infer that low intrinsic value could usually induce low enjoyment, although their perceived control and perceived extrinsic value were high. It suggests that high intrinsic value was closely related to high enjoyment. Besides, it also indicates that intrinsic value is predominant in eliciting achievement emotions.

Pride was closely associated with perceived control. By comparing participants 1, 5 and participant 6 (different in perceived control), we could infer that although low perceived intrinsic value could induce fewer positive emotions and more negative achievement emotions, the specifically elicited achievement emotions were also interactively determined by perceived control. With high extrinsic value and low intrinsic value, high perceived control might induce fear, but hope was probably still high. With high extrinsic value and low intrinsic value, anguish might be elicited, but pride was probably still high. It suggests that high perceived control was closely related to high pride.

Hope seems to have generally existed since five out of six participants were reported to have high hope. And hope seems largely independent of control-value appraisals because by comparing participants 1, 5, and participants 2, 3, and 4 (only the same in extrinsic value), hope does not seem to be related to perceived control and perceived intrinsic value but related to perceived extrinsic value. However, by further comparing participants 1, 5, and participant 6 (same in extrinsic value), participant 6’s low hope indicates that hope does not seem to be related to perceived control as well.

#### Association between achievement emotions and EFL performance

According to the CVT, achievement emotions can also have an impact on academic performance. Therefore, we made Table 4 comprising the participants’ achievement emotions and their English grades in the final exam. Under the framework of the CVT, the analysis was based on the comparison of the similarities and differences between the variables.

**Table 4:** Participants’ achievement emotions and English grades.

Participant	Positive emotions	Negative emotions	Grade
1	Fewer	Fear	72.5
2	More	/	118.5
3	More	/	116
4	More	/	112
5	Fewer	Fear	76
6	Fewer	Anguish	116

By comparing participants 2, 3, and 4 (more positive emotions) and participants 1 and 5 (fewer positive emotions and more negative emotions), we could infer that more positive achievement emotions (enjoyment, pride, and hope) were associated with better EFL performance, whereas more negative achievement emotions were associated with worse EFL performance. Nevertheless, by comparing participant 6 and participants 2, 3, and 4, we found that worse EFL performance does not seem to be the corollary of negative achievement emotions. In the first interview, participant 6 gave the reason that her past high English grade in her junior high school contributed to her current high English grade in her senior high school. Therefore, we could infer that past EFL achievement could have a positive impact on current EFL performance and counterbalance the negative impact of negative achievement emotions on current EFL performance.

### Other antecedents of achievement emotions

In order to provide empirical evidence for emotional intervention in Chinese senior high school students’ EFL learning, we also explored these Chinese high school students’ other antecedents of EFL learning in addition to control-value appraisals. According to the analysis of the data from the interviews and the focus group discussion, three themes emerged, namely factors inside the classroom, factors outside the classroom, and personal factors (see Table 5).

**Table 5:** Other antecedents of achievement emotions.

Code	Sub-theme	Theme
Teaching methods	Teachers	Factors inside the classroom
Character		
Attitude toward students		
Values		
Appearance		
	Classmates	
	Equipment	
	Social values	Factors outside the classroom
	Parental expectations	
	Interest	Personal factors
	Career goals	

#### Factors inside the classroom

Factors inside the classroom consisted of three sub-themes: teachers, classmates, and equipment. EFL teachers were found to play a crucial role in arousing positive achievement emotions in EFL learning. More specifically, it involved the teacher’s teaching method: *“Her diverse teaching methods, intelligible teaching language and relaxing learning atmosphere make me engrossed and enjoyable in English learning”* (Participant 1—Focus group discussion); character: “*her great character is so contagious. She’s quite outgoing, easygoing, and humorous”* (Participant 3—Focus group discussion); attitude toward students: *“Every time I asked her questions, she would always be patient to answer them. It is her attitude that wins our favor and makes us relaxed and pleasant”*. (Participant 1—Focus group discussion); values: *“She often shares her life experiences and views on social issues, which, I think, helps us shape our correct values”* (Participant 4—Focus group discussion); and appearance: *“I’m fond of this teacher, because my impression of her is comely and lovely”* (Participant 6—Focus group discussion).

Classmates were also found to affect achievement emotion in EFL learning. For example, “*I hope those introverted classmates could have been more active in English class, so that both the teacher and we the classmates would not feel so bored”* (Participant 4—Focus group discussion). Besides, equipment was found to be one of the factors since it was frequently grumbled by the participants: “*Every time our English teacher tries to open a video, the computer just does not work. It makes us bitterly sad”* (Participant 5—Focus group discussion).

#### Factors outside the classroom

Factors outside the classroom had two sub-themes: social values and parental expectations. As for social values, all participants held the belief that society is friendlier to people with academic achievement. Most people attached importance to English and EFL learning, as English is one of the key subjects to be examined in Gaokao. Participants 2, 4, and 6 embraced this belief and one of them mentioned: *“I think a person’s great academic performance could prove his ability in learning. Learning ability is emphasized by this society. Therefore, those with great academic performance would be treated with greater leniency and friendliness in this society”* (Participant 2—Focus group discussion). Participants 1, 3, and 5 thought that apart from academic performance, one’s EQ, habit, style to deal with matters, and character are all important to a person. However, by reading between the lines, it could be inferred that when other things are equal, only the students with better performance would stand out, implying their unconscious agreement on the view that Chinese culture belongs to a performance-valued society.

Parental expectations were also found to affect the value of EFL learning, as one participant mentioned: “*My parents hope I should try my utmost to get the laurels. That’s why I must improve my weak subjects like English. Their expectations spur me to do what I’m not good at. When I get encouragement from them, I would be more joyful and passionate to memorize and recite English-related materials”* (Participant 5—Focus group discussion).

#### Personal factors

Personal factors also included two sub-themes: interest and career goals. Interest in English was repeatedly mentioned to play a significant role in the arousal of pleasant feelings in the focus group discussion. For example, one participant mentioned: “*I have great interest in English, so I plan to have a job using English. I’m very devoted to learning English because I love it”* (Participant 4—Focus group discussion).

Career goals were also found to be closely related to hope in the second interview. Participant 6 told us that she held a pessimistic attitude toward her English learning because she did not think she would use English: she would be a medicine major after the College Entrance Exam. From her remarks, we could infer that apart from the control-value appraisals, career goals could also greatly affect hope. When English is subjectively judged to be irrelevant to career goals, hope in EFL learning would be diminished.

## Discussion

Based on the semi-structured interviews and the focus group discussion, we examined six Chinese senior high school students’ control-value appraisals, achievement emotions, and EFL performance, and then analyzed their relationship under the framework of the CVT. Besides, we also explored other antecedents in addition to control-value appraisals. Regarding research question 1, we found that high perceived control and high perceived extrinsic and intrinsic values were interactively associated with enjoyment, pride, and hope. Low perceived control, high perceived extrinsic value, and low perceived intrinsic value were interactively associated with fewer positive achievement emotions (only hope) and more negative achievement emotions like fear. High perceived control, high perceived extrinsic value, and low perceived intrinsic value were interactively associated with fewer positive achievement emotions (only pride), and more negative achievement emotions like anguish. Specifically, enjoyment was closely associated with perceived intrinsic value, whereas pride was closely associated with perceived control, which was consistent with the CVT which proposes that enjoyment depends on a combination of positive competence appraisals and positive intrinsic value appraisals, and pride relies on perceived control (specifically, perceived control of success or failure; Pekrun and Perry, 2014). However, different from the CVT which assumes that hope was instigated by the uncertainty of control, we found that the hope among the six participants seemed independent of control-value appraisals, which might be justified by traditional Chinese culture. In a Confucian society, when pursuing academic goals, Chinese students have the belief that effort can conquer one’s limitations and improve one’s academic performance. This belief would lead Chinese students to attribute their academic success or failure to their effort, irrespective of control-value appraisals. In other words, in China, the proximal determinant of hope in academic settings is probably students’ belief in efforts rather than their control-value appraisals (Hau and Ho, 2012; Chen et al., 2018).

As for research question 2, we found that more positive achievement emotions (enjoyment, pride, and hope) were positively associated with EFL performance while negative achievement emotions were negatively associated with EFL performance, which was in line with the assumptions of the CVT (Pekrun and Perry, 2014) that enjoyment, pride, and hope can exert a positive effect on learning and performance, as well as in line with the conclusion of Shao et al. (2020b) that enjoyment, pride, and hope can have a positive influence on EFL performance. It is noteworthy that we found that negative achievement emotions were not necessary to be associated with worse EFL performance. Current EFL performance would also be influenced by prior EFL performance. The positive effect of prior excellent EFL performance on current EFL performance could offset the negative effect of negative achievement emotions on current EFL performance.

Concerning research question 3, we found three kinds of antecedents of Chinese high school students’ achievement emotions in EFL learning, namely factors inside the classroom, factors outside the classroom, and personal factors. Among factors inside the classroom, teachers were salient, as teachers experienced in teaching methods, nice in character, warm in attitude toward students, sophisticated in values, and easy-going in appearance, could instigate students’ positive emotions, particularly enjoyment. This was consistent with previous studies (Dewaele et al., 2018, 2019; Li, 2022), in which, teachers were found to play a pivotal role in inducing enjoyment in EFL learning. Besides, classmates’ engagement could also affect students’ achievement emotions in EFL learning. In detail, we found that unengaged classmates could trigger boredom, which was in line with the studies of Chapman (2013) and Nakamura et al. (2021), in which it was found that classmates’ unengaging behaviors could create a discouraging atmosphere, consequently inducing boredom. In addition, equipment in the classroom could also affect students’ achievement in EFL learning. Old and unresponsive equipment was found to trigger students’ negative achievement emotions in EFL learning. Factors outside the classroom include parental expectations and social values. We found that parents’ expectations of children’s academic achievement and society’s value of people with academic achievement could make students attach more importance to EFL learning, thus influencing their control-value appraisals and achievement emotions. This was in line with the assumptions of the CVT (Pekrun and Perry, 2014) that important people’s subjective expectations and social values are vital factors in influencing achievement emotions. Personal factors include interest and career goals. The former was also recognized in the CVT. As for the latter, we found that it was closely associated with hope—if English was related to students’ career goals, then he or she would probably gain higher hope. If not related, then lower hope. Combined with the great effect of the belief in efforts on hope mentioned above, we assume that hope is a complicated and culture-related positive emotion that deserves to have further studies.

## Implications

The educational implications of our study are as follows. First of all, it is advisable for Chinese high school English teachers to increase students’ perceived control and perceived intrinsic value. Attribution training can be adopted by teachers to enhance students’ perceived control. Specifically, it is appropriate to lead students to attribute both their success and failure in EFL learning to their effort instead of their competence, thus increasing their confidence and decreasing their sense of uncontrollability (Weiner, 1985, 1992, 2007). Besides, teachers could employ a hierarchical teaching model to effectively stimulate the perceived intrinsic value of EFL learning (Yang, 2018). Secondly, considering the significance of emotions in affecting EFL performance and in order to induce enjoyment and reduce boredom, diverse teaching methods like emotion-based language instruction (Pishghadam et al., 2013) and connectivism instructional method (Borna and Fouladchang, 2018) are advisable to be adopted by English teachers in the English class to motivate students’ English learning by emotionalizing language learning and reducing their negative emotions. Besides, it is reasonable for teachers to keep outgoing, humorous, friendly, and neatly dressed when getting along with students. Thirdly, schools should invest more funding to update the old equipment to guarantee teaching activities. Fourthly, teachers are encouraged to devote themselves to the construction of positive cultural values of this society, and parents are expected to place certain expectations on their children.

Our study has several novelties. Firstly, this study, to our knowledge, is the first one that qualitatively investigated the relationship between Chinese high school students’ control-value appraisals, positive achievement emotions (enjoyment, pride, and hope), and EFL performance. We found hope is independent of control-value appraisals and demonstrated the CVT’s cross-cultural applicability to a great extent. In addition, we further explored and found some other antecedents of achievement emotions in addition to those in the English classroom, thus providing a theoretical reference for an overall intervention in achievement emotions of EFL learning. However, our study has also some limitations. In the first place, although qualitatively acceptable, the number of participants in our study is very small (Mackey and Gass, 2016). In the second place, we focused on only three positive achievement emotions (enjoyment, pride, and hope) and failed to incorporate other positive ones like relaxation into our study. In the third place, although the analysis was under the framework of the CVT, the causality between control-value appraisals, achievement emotions, and EFL performance might be less compelling than regression analysis. Future studies can be conducted using mixed methods of qualitative and quantitative approaches with the expansion of sample size and the inclusion of more positive achievement emotions to examine the causality between these variables.

## Data availability statement

The original contributions presented in the study are included in the article/supplementary material, further inquiries can be directed to the corresponding author.

## Ethics statement

The studies involving human participants were reviewed and approved by Jilin International Studies University. Written informed consent for participation was not required for this study in accordance with the national legislation and the institutional requirements. Written informed consent was not obtained from the individual(s) for the publication of any potentially identifiable images or data included in this article.

## Author contributions

WY: supervision, conceptualization, methodology, and writing–reviewing, revising, and editing. HW: conceptualization, methodology, formal analysis, and writing–original draft and revising. WZ: conceptualization, methodology, study design, and data collection. All authors contributed to the article and approved the submitted version.

## Conflict of interest

The authors declare that the research was conducted in the absence of any commercial or financial relationships that could be construed as a potential conflict of interest.

## Publisher’s note

All claims expressed in this article are solely those of the authors and do not necessarily represent those of their affiliated organizations, or those of the publisher, the editors and the reviewers. Any product that may be evaluated in this article, or claim that may be made by its manufacturer, is not guaranteed or endorsed by the publisher.
